# A synthesis of qualitative evidence of barriers and facilitators in implementing guidelines for TB testing in healthcare settings

**DOI:** 10.1186/s43058-024-00565-0

**Published:** 2024-03-27

**Authors:** Perpetua W. Karanja, Mercy N. Mulaku, Eleanor A. Ochodo

**Affiliations:** 1 Department of Health, Kirinyaga County, Kerugoya, Kenya; 2 Centre for Evidence-Based HealthCare, Division of Epidemiology and Biostatistics, Faculty of Medicine and Health Sciences, Stellenbosch University, Stellenbosch, South Africa; 3 Centre for Global Health Research, Kenya Medical Research Institute, Nairobi, Kenya; 4 Department of Pharmacology, Clinical Pharmacy, and Pharmacy Practice, Faculty of Health Sciences, University of Nairobi, Nairobi, Kenya

**Keywords:** Tuberculosis, Testing guidelines, Algorithms, Tuberculosis testing, Barriers, Facilitators

## Abstract

**Introduction:**

The suboptimal case notification rates for tuberculosis (TB) globally could partly be due to the poor implementation of TB testing guidelines or policies. We identified, appraised and synthesized qualitative evidence exploring the barriers and facilitators to implementing TB testing guidelines.

**Methods:**

We searched electronic databases and grey literature and included studies based on predefined inclusion criteria (PROSPERO registered protocol CRD42016039790) until 9th February 2023. We used the Critical Appraisal Skills Programme tool to assess the methodological quality of the included studies. Two authors reviewed the search output, extracted data and assessed methodological quality independently, resolving disagreements by consensus. We used the Supporting the Use of Research Evidence framework to identify themes and analyse and synthesize our data. We applied the Confidence in the Evidence from Reviews of Qualitative Research approach to assess the confidence of the review findings.

**Results:**

Our search output was 6976 articles, from which we included 25 qualitative studies, mostly from low- and middle-income countries (*n*=19) and about national guidelines (*n*=22). All studies were from healthcare settings. Most barriers revolved around health system constraints involving the guidelines (low trust and adherence, ambiguous and poorly developed or adapted guidelines) and poorly resourced and organized health facilities to enable the implementation of the guidelines. Individual-level barriers included low trust and low awareness among recipients and providers of care. Donor dependence was the main socio-political constraint. These barriers were similar across all income settings except poorly resourced health facilities and social and political constraints which were only reported in low- and middle-income settings. The reported facilitators were improved trust and knowledge of guidelines, national leadership support and availability of training tools and opportunities for guidelines across all income settings. We had high confidence in most of the review findings.

**Conclusion:**

Limited guideline knowledge, trust and adherence related to poorly developed and disseminated guidelines in all income settings and poorly resourced facilities in low- and middle-income countries hinder the implementation of TB testing guidelines. This could be improved by better guideline training and adaptation and resourcing of health facilities.

**Trial registration:**

The protocol of this review was registered with the International Prospective Register of Systematic Reviews (PROSPERO), registration number CRD42016039790, and published in a peer-reviewed journal.

**Supplementary Information:**

The online version contains supplementary material available at 10.1186/s43058-024-00565-0.

Contributions to literature

1. Poorly developed guidelines, inadequate awareness about TB disease and guidelines and poorly resourced health facilities were the main barriers to TB testing guideline implementation.
2. Patients’ willingness to test, adequate knowledge about guidelines and availability of tools and training opportunities promoted the implementation of the TB testing guidelines.
3. There was scarce evidence on barriers and facilitators of implementing TB testing guidelines in community or home settings and minimal evidence from high-income settings.

## Background

Tuberculosis (TB) remains a major cause of ill health and one of the leading causes of death from a single infectious agent. In 2022, there were 1.3 million TB-related deaths globally. The net reduction in TB incidence and mortality from 2015 to 2022 was 8.7 and 19%, respectively, which were far below the targets set by the End-TB strategy of a 50% reduction in incidence and a 75% reduction in mortality by 2025 [1].

The World Health Organization (WHO) routinely recommends and introduces guidelines and tests to improve the bacteriological diagnosis of TB; however, the detection of new cases remains suboptimal. In 2022, the bacteriologically confirmed cases of pulmonary TB were only 63% globally. Detection of drug resistance TB relies on testing for drug resistance using culture methods, rapid molecular tests and sequencing technologies to ensure that patients receive appropriate drugs on time. Only 73% of bacteriologically confirmed TB cases were tested for drug resistance in 2022 [1]. The emergence of coronavirus disease 2019 (COVID-19 pandemic) affected the detection of new cases in 2021 globally with the disruptions mainly associated with stigma due to similarities in symptoms and reallocation of resources to the COVID-19 response [2].

Poor case detection of TB can be caused by poor implementation of guidelines to diagnose TB. For example, a trial assessing health worker adherence to TB diagnostic algorithms revealed poor adherence to the algorithms or guidelines [3]. Guidelines support healthcare workers in identifying patients with the disease using recommended tests and diagnostic algorithms and assist in facilitating the implementation of a recommended test. The most available evidence about poor adherence to recommended policy guidelines and diagnostic algorithms is from quantitative study designs [3–5]. Factors related to healthcare stakeholders (healthcare workers, patients and managers among others), the health system and contextual factors are some of the reasons cited by primary studies for poor implementation of guidelines [6–9].

Qualitative evidence can comprehensively provide insights and perspectives into why implementation strategies for TB testing guidelines succeed or fail from the perspectives and experiences of stakeholders which may not be comprehensively captured by quantitative evidence. Some qualitative systematic reviews on the implementation of TB control strategies [10] or individual TB tests [11] and a mixed methods review on diagnosis and treatment of drug-resistant TB [12] are available but they do not explicitly focus on implementation of guidelines for TB testing. There could be differences in considerations or perspectives for implementing the guideline itself versus focusing on the individual test or TB control strategies in general. For example, the guideline itself could be poorly developed, ambiguously presented or poorly disseminated [10–13].

To our knowledge, there is no qualitative systematic review exploring factors influencing the implementation of diagnostic guidelines for TB from the perspectives or experiences of healthcare stakeholders. The information will guide the implementation of TB diagnostic guidelines and design interventions to improve the uptake of guidelines. We, therefore, identified, appraised and synthesized qualitative evidence to explore the barriers and facilitators to implementing guidelines on TB diagnosis from the perspectives of healthcare stakeholders.

## Methods

The protocol of this review was registered with the International Prospective Register of Systematic Reviews (PROSPERO), registration number CRD42016039790, and published in a peer-reviewed journal [14]. Changes to the protocol have been outlined in Additional file 1. We have reported this qualitative review according to the Enhancing Transparency in reporting the synthesis of qualitative research ENTREQ statement and the Preferred Reporting Items for Systematic Reviews and Meta-analysis (PRISMA) checklist (Additional files 2 and 3) [15, 16].

### Criteria for considering studies for this review

We included studies that fulfilled the following criteria:

#### Types of studies

We included primary studies that employed qualitative methodology following guidance from the Cochrane Handbook for Systematic Reviews [17], which defines “a qualitative study as a study that uses a qualitative method of data collection and analysis”. We therefore included studies that used both qualitative methods for data collection such as individual interviews and focus group discussions and qualitative methods for data analysis such as thematic analysis, and grounded theory. For studies that used mixed methods, we included only data that used qualitative methods for data collection and data analysis. We excluded studies that collected data using qualitative methods but analysed the data quantitatively. We excluded studies with comments from quantitative surveys, editorials, conference abstracts and opinion pieces.

#### Types of participants

We included studies that report on the perspectives of health workers, health managers, policymakers, patients, activists, academics and other stakeholders that we came across in the studies towards implementing guidelines for TB diagnosis. We defined health workers as “all people engaged in actions whose primary intent is to enhance health”, as recommended by the WHO [18].

#### Type of setting

We included studies from any geographical setting globally and any setting where TB diagnosis is conducted, including healthcare facilities, the community and during home visits.

#### Types of interventions

We applied a broad definition of the term guideline described as “systematically developed statements to assist practitioner and patient decisions about healthcare for specific clinical circumstances” [19]. A guideline could also be referred to as a policy, protocol or algorithm [19]. We included any qualitative study that explored the implementation of any guideline, whether it was the main focus of the study or nested within the study.

We included studies that focused on implementing a guideline about any test, for any form of TB, including latent TB infection, pulmonary or extrapulmonary disease, and drug-susceptible or drug-resistant disease. A TB test could refer to a screening or diagnostic test. We also included studies that assessed guideline implementation strategies or interventions. Examples of guideline implementation strategies or interventions included those targeted at healthcare organizations (organizational culture, continuous quality improvement), healthcare workers (education, training, audit and feedback, reminders, patient-mediated interventions), and patients (reminders, financial incentives) [20].

#### Types of outcome measures

The phenomena of interest in this review were attitudes, perspectives and experiences of health stakeholders (for example, health workers, managers, policymakers and patients) when implementing guidelines on TB diagnosis.

### Search methods for the identification of studies

We developed a search strategy using guidelines recommended by the Cochrane Qualitative Research Methods group [21] and searched multiple electronic sources from inception until 4th February 2020 with updated searches on 13th Dec 2021 and on 9th February 2023.

The search strategy incorporated the key terms: “guidelines”, “tuberculosis”, “implementation”, and their associated synonyms. We searched electronic databases, including MEDLINE, EMBASE, TRIP, The Cochrane Library, Cumulative Index to Nursing and Allied Health Literature (CINAHL) and several regional databases (African Index Medicus, Index Medicus for the Eastern Mediterranean Region, INDMED, HERDIN, Thai Index Medicus, LILACS). The detailed search strategies can be found in Additional file 4.

We also checked for searches from conference proceedings within our search output and searched reference lists of the selected relevant studies. The search output was collated into an EndNote™ file [22] and imported into the systematic review platform Eppi Reviewer™ [23].

### Study selection

We used Eppi Reviewer™ [23] to screen titles, abstracts and full texts of the search output. To minimize selection bias, PWK, MNM and EAO independently screened the search outputs for potentially eligible studies in parallel, compared their selections, and resolved disagreements by discussion and consensus. Thereafter, PWK, MNM and EAO independently screened the full text of potentially eligible articles to check if the articles fulfilled the inclusion criteria defined by the types of studies, participants, intervention, setting and outcomes. The search results are presented in a flow diagram recommended by PRISMA [15].

### Data collection and analysis

Drawing from the Supporting the Use of Research Evidence (SURE) framework [24], we developed a structured and standardized data collection form for extracting data from the selected studies. The SURE framework focuses on barriers to implementing health systems interventions and includes elements on knowledge and skills, health system challenges and social and political constraints (Additional file 5). To ensure the integrity of the assessment, PWK and EAO piloted the data collection form on at least three studies identified from the list of potentially eligible studies. We extracted data about the first author, publication year, journal, language, participant group (cadre of health workers), setting (country, rural/urban, type of health facility), intervention (type, description and recommendation of the guideline, the test, test strategy or algorithm and form of TB focused on by the guideline), research methods (method of data collection and analysis, framework used) and outcomes (reported barriers and facilitators and related themes). PWK, MNM and EAO independently and in parallel extracted the data using the Eppi Reviewer™ platform and resolved disagreements through discussion.

We categorized studies by income category using the World Bank income classification of countries [25]. The studies were classified as low income (LI), low middle-income (LMI), upper-middle-income (UMI) and high-income (HI) countries. Low- and middle-income countries (LMICs) included the low-income and middle-income countries.

### Assessment of the quality of the included studies

We used an adaptation of the Critical Appraisal Skills Programme (CASP) quality assessment tool for qualitative studies to assess the methodological quality of the included studies [26]. PWK, MNM and EAO independently and in parallel applied the CASP tool (Additional file 6) and resolved disagreements through discussion. Our CASP checklist had ten questions, which were scored as either Yes, No or Unclear. We did not use the assessments of methodological quality to exclude studies but to assess how much confidence we have in each finding.

### Data synthesis

We used the thematic framework analysis approach to analyse and synthesize qualitative data drawing on the SURE framework [27, 28]. Thematic synthesis is useful where the evidence is likely to be primarily descriptive and enhance our understanding of why health stakeholders think, feel and behave as they do.

The first author (PWK) began by familiarizing herself with the data against the review’s aims and noted recurrent themes across the studies. We then used the SURE framework to guide our thematic analysis across five main domains: recipients of care level, providers of care level, other stakeholders’ level, health systems and socio-political constraints. We also included other emerging themes from our analysis. We read all the studies until there were no new emerging themes. We then coded the data based on the themes identified in the data, indexing using the codes related to the themes of the framework. We indexed some studies with one or more codes.

We sorted the data by themes and presented the themes in the form of an analysis table, enabling us to summarize the findings of the studies across different themes and subthemes. We then mapped and interpreted our results in line with the review objectives and emerging themes and explored associations between the themes to help better explain the findings. Data coding and charting were performed by PWK and double-checked by EAO or MNM with disagreements resolved through discussion.

### Assessment of confidence in the review findings

We applied the Confidence in the Evidence from Reviews of Qualitative Research (CERQual) approach to explain and summarize our judgements on the confidence of the systematic review [27]. This approach draws on the principles of the Grading of Recommendations, Assessment, Development and Evaluation (GRADE) approach. The CERQual approach assesses confidence in the review findings based on four components: the methodological limitations of the included studies, the relevance of the included studies to the review question, the coherence of the review findings and the adequacy of the data contributing to the review findings.

PWK applied the GRADE CERQual approach to assess the confidence in each of the review findings and discussed the judgements with EAO and MNM who confirmed or modified the judgements. After assessing each of the four components, we all made a judgement about the overall confidence of the findings on consensus by discussion. Based on our assessment, we judged the overall confidence in the review findings as high, moderate, low or very low. The starting point of high confidence suggests that the review finding is highly likely a reasonable representation of the phenomena of interest. We presented this assessment in a summary of qualitative findings table that includes not only the judgement “high”, “moderate”, “low” and “very low” but also a written justification for the assessment.

## Results

### Search results

**Fig. 1:**
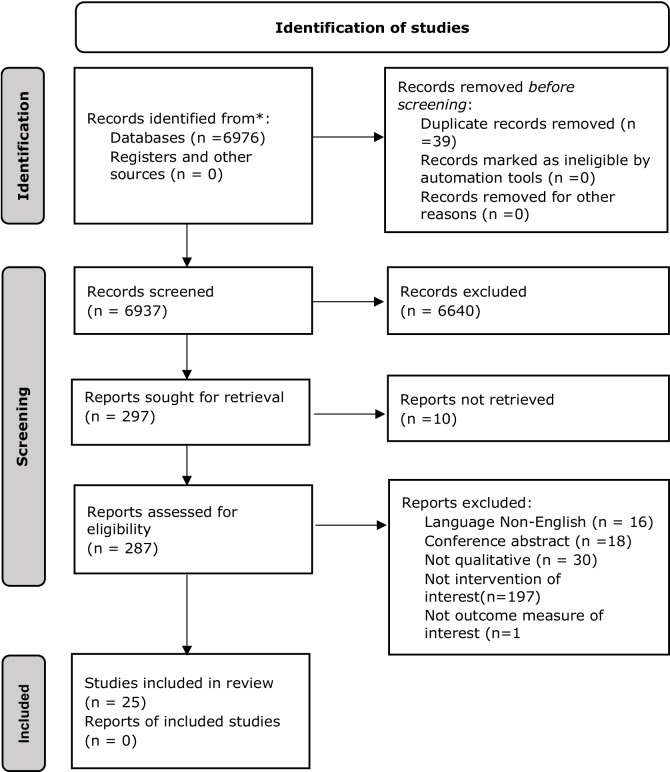
PRISMA flow diagram

Our search identified 6976 articles from electronic and grey literature searches (Fig 1). We screened 6937 titles and abstracts after duplicates were removed. We excluded 6640 titles and abstracts and screened 287 full-text articles. Ten full texts were inaccessible [*n*=10]. We excluded 262 full-text articles. Reasons for exclusion included non-English articles [*n*=16], conference abstracts [*n*=18], non-qualitative study design (did not use qualitative methods for analysis) [*n*=30], ineligible intervention [*n*=197], and ineligible outcome measures [*n*=1]. We included 25 articles in the qualitative synthesis.

### Description of included studies

A summary of the study characteristics can be found in Table 1. Of the 25 studies, 24 mentioned data about a guideline or policy (main or nested focus), while one Siddiqi et al. [29] was on an implementation strategy (clinical audit). Three studies explored global policies/guidelines [8, 29, 30], while 22 papers evaluated the implementation of national policies/guidelines. A list of excluded studies is shown in Additional file 7.

**Table 1:** Characteristics of the included studies

**Author (year)**	**Country**	**Income category**	**Setting**	**Participants**
Chadha 2014 [31]	India	LMI	Not reported	Medical officers
Evenblij 2016 [32]	Netherlands	HI	Public HIV treatment centres	Physicians
Farhoudi 2017 [9]	Iran	LMI	Public prison facility outside the city	Prison healthcare officers, physicians, infectious disease specialists, experts on jail diseases and prison governors
Jia 2016 [33]	China	UMI	Rural and urban outpatient and inpatient settings	CDC TB heads, insurance managers, healthcare managers and healthcare workers
Joseph 2004 [8]	USA	HI	Rural and urban health departments and hospitals	Clinical, janitorial, administrative and clerical staff
Kerrigan 2018 [34]	South Africa	UMI	General primary healthcare clinics	Patients and healthcare workers
Mala 2014 [35]	Ethiopia	LI	Public out and inpatient settings	TB service providers
McDowell 2018 [36]	India	LMI	Urban public and private health settings	Physicians
Rendell 2017 [37]	Mongolia	LMI	Public out and inpatient settings	Healthcare workers and administrative staff
Siddiqi 2008 [29]	Cuba, Peru, Bolivia	UMI and LMI	Peri-urban primary care settings	Healthcare workers
Spruijt 2019 [38]	Netherlands	HI	Public health service settings	TB service providers
Wei 2010 [39]	China	UMI	Public out and inpatient settings	TB suspects, Hospital managers and healthcare workers
Nagar 2020 [40]	India	LMI	Public and private out and inpatient settings	Orthopaedic surgeons/physicians
Mulder 2012 [7]	Netherlands	HI	Public outpatient health facilities	Public health nurses
Biermann 2020 [30]	Global	UMI, LMI and LI	Not reported	Stakeholders involved in ACF policy development and implementation
Biermann 2020 [5]	30 high TB-burden countries	LMI, UMI and LI	Not reported	National TB Programme (NTP) managers
Singh 2021 [41]	India	LMI	Not reported	TB service providers
Nalugwa 2020 [42]	Uganda	LI	Public out and inpatient settings	Front-line Study staff, field notes from study staff
Oliwa 2020 [43]	Kenya	LMI	Out and inpatients	TB service providers
Naidoo 2015 [6]	South Africa	UMI	Public outpatient health facilities	Patients
Mwaura 2021[44]	Kenya, Uganda, and South Africa	LMI, LI and UMI	Private and public facilities	TB service providers, programme officers, and patient advocates
Kuznetsov 2016 [45]	Russia	UMI	Public outpatient health facilities	Directors and managers of facilities and TB service providers
Kanakaraju 2020 [46]	India	LMI	Public out and inpatient settings	TB service providers
Gray 2022 [47]	United Kingdom	HI	Public inpatient and outpatient (hospital-based)	Non-public health clinicians: consultants in respiratory medicine and infectious disease Public Health clinicians TB clinical nurse Specialist
Szkwarko 2022 [48]	USA	HI	Public and Private facilities Outpatient settings	Primary Care Providers: Physicians, physician’s assistants, and nurse practitioners.

*LI* Low income, *LMI* Lower middle income, *UMI* Upper middle income, *HI* High income, *CDC* Centers for Disease Control and Prevention, *ACF* Active case finding

Eight studies were from lower-middle-income countries [9, 31, 36, 37, 40, 41, 43, 46], five were from upper-middle-income countries [6, 33, 34, 39, 45], six were from high-income countries [7, 8, 32, 38, 47, 48] and two were from low-income countries [35, 42]. Four studies had representatives from a mix of countries with different income levels [5, 29, 30, 44]. All studies were conducted in health care settings. Participants ranged from patients, patient advocates, healthcare workers, TB programme managers at various levels, policymakers and Centers for Disease Control (CDC) heads.

Various data collection methods were used in the studies; nine used semistructured interviews, seven used mixed methods, six used in-depth interviews, two used focused group discussions and one used observation methods.

### Quality of included studies and confidence in review findings

In general, there was poor reporting of reflexivity and methodological theory across the included studies in the CASP quality assessment tool. All studies reported about the sampling, data collection and data analysis methods. Only 18 of the studies adequately reported the study setting [6–9, 29, 32, 34–40, 42, 44, 46–48]. The CASP assessment results are presented in Additional file 8.

Using the CERQual approach for the identified 55 review findings, we graded 38 review findings as high confidence, 13 review findings as moderate confidence and 4 review findings as low confidence. The quality judgement for each finding is summarized in Additional files 9 and 10.

## Themes

We summarized the main barriers and facilitators to the implementation of TB diagnosis guidelines in Table 2 and categorized them using the SURE framework levels and domains. Themes about barriers were more prominent in the included studies compared to the facilitators. Most themes were reported in both low- and middle-income countries (LMICs) and high-income (HI) countries except for poor-resourced facilities and those in the social and political constraints level which were only reported in LMICs. A comprehensive list of all barriers and facilitators is summarized in Additional files 11 and 12.

**Table 2:** Barriers and facilitators of implementing TB testing guidelines implementation (main findings)

**Level**	**SURE framework domains**	**Barriers**	**Facilitators**
Recipients of care	Attitudes regarding programme acceptability, appropriateness and credibility	Mistrust of tests and health providers Disbelief of diagnosis Stigma	Trust in doctors and willingness to test
Providers of care	Knowledge and skills	Limited awareness of guidelines Poor understanding of disease Limited skills in sample collection	Knowledge about disease and guidelines
	Attitudes regarding programme acceptability, appropriateness and credibility	Lack of confidence in tests Mistrust of guidelines Fear of misusing tests Patient’s reaction guiding practice	
Social and political constraints	Donor policies	Donor and WHO dependence Low national policy ownership	
	Influential people		National leadership support Political will Stakeholder engagement
Health systems	Relationship with norms	Low adherence to guidelines Ambiguous guidelines inflexible guidelines Poorly updated guidelines Poor advocacy Rigorous guidelines methodology Poorly adapted guidelines	
	Education system	Limited education	Training tools and opportunities
	Patient flow and processes	Poor patient flow process	
	Facilities	Poor facilities Logistical and specimen transport issues	

For both the recipients of care and providers of care levels, themes related to attitudes regarding programme acceptability, appropriateness and credibility domain were the most prominent. The knowledge and skills domain was also crucial for providers of care. Donor policies and influential people domains were commonly reported for the social and political constraints levels. Themes related to health system constraints were mainly reported. Under the health system constraints level, relationships with norms, education system, facilities and patient flow and processes domains were commonly reported.

### Recipients and providers of care level

#### Attitudes regarding programme acceptability, appropriateness and credibility

Among the recipients of care (patients and caregivers), mistrust of the TB diagnostic tests and healthcare providers and stigma about TB disease were barriers to implementing TB diagnostic policies and guidelines [8, 29, 34, 38, 40]. Patients were concerned about the TB tests’ validity, were likely to refuse tests prescribed in the guidelines and lacked confidence in the healthcare providers. Patient-level barriers were reported across both LMICs and HI countries. Patients’ beliefs and attitudes about TB disease influenced TB guidelines and policy applicability. In contrast, patients’ trust in doctors and their willingness to test facilitated the implementation of the guidelines for both LMICs and HI countries [5, 8, 46].

For providers of care, lack of confidence in TB tests, mistrust of the guidelines, fear of misusing tests, perceived difficulty in obtaining specimens and patients’ reaction guiding practice were barriers to implementing TB diagnostic guidelines [6, 29–32, 34–36, 40, 41, 43–45, 47, 48]. Lack of confidence in TB test factors were perceived non-feasibility, low sensitivity of diagnostic tests and scepticism about test speed and reliability [6, 29–32, 34–36, 40, 41, 43–45, 47, 48]. Providers deviated from guidelines/algorithms based on their perceptions of patient reactions to clinical decisions, for example, ordering X-rays against clinical policy to meet perceived expectations of the patient [29]. All barriers were reported across both LMICs and HI countries. The potential to reduce future workload promoted the implementation of guidelines in both LMICs and HI countries [47].

#### Knowledge and skills

For providers of care, barriers to implementing TB diagnosis guidelines were poor awareness and understanding of guidelines/diagnostic algorithms, poor understanding of TB disease and challenges in sample collection, especially among children [6–8, 31, 34, 36, 37, 39–41, 43, 44, 47, 48]. Adequate knowledge about the guidelines/diagnostic algorithms and disease promoted the implementation of TB diagnostic guidelines [5, 6, 36, 37, 40, 43, 45, 46, 48]. Both knowledge and skills barriers and facilitators were reported in both LMICs and HI countries.

### Social and political constraints level

Overdependence on donor funding and lack of country ownership of the policies were barriers to TB diagnostic guideline implementation in LMICs. The donor’s influence in implementation and setting targets for funding recipients affected ownership of the TB policies [30]. National TB leaders’ support, political will and stakeholder engagement at different levels of care were drivers of the implementation of TB diagnostic guidelines in LMICs [5, 30].

### Health system constraints level

Barriers related to guideline quality and development were prominent in the included papers: ambiguous guidelines, poorly updated guidelines, rigorous guideline methodology and poorly adapted guidelines. In addition, poor advocacy of guidelines and low adherence to guidelines deterred the implementation of TB diagnostic guidelines and were reported in both LMICs and HI countries [6, 7, 29, 35, 37–41, 44]. The ambiguity in guideline recommendations was due to guidelines being nonspecific to certain subgroups of patients. Poor adherence to guidelines was linked to overdiagnosis of patients [7].

Limited education on guidelines and TB disease was a barrier to TB diagnostic guideline implementation across all settings [6, 29, 36, 37, 39, 41, 43, 45, 48]. The availability of tools (guidelines, algorithms and care pathways) and training opportunities, such as workshops, practical and face-to-face training sessions, and internet modules, facilitated the implementation of TB diagnostic guidelines and were reported in both LMICs and HI countries [8, 9, 29, 31, 34, 37, 40, 41, 45]. Poor patient flow processes were also a significant barrier to the implementation of TB diagnostic guidelines in both LMICs and HI countries [6, 8, 30, 31, 35, 43, 46].

Poor facilities, such as insufficient tests and infrastructure and logistical and poor specimen transport issues, were crucial barriers to TB diagnostic guideline implementation in LMICs [6, 35, 39, 41, 42, 45, 46]. The COVID-19 pandemic disrupted the implementation of guidelines as TB screening services were paused and priority was given to the COVID-19 pandemic and was reported in a HI country [47].

## Discussion

Our review aimed to identify barriers and facilitators in implementing guidelines for the diagnosis of TB guided by the SURE framework which provided domains to describe the implementation of health interventions and policies systematically. Most included studies were from LMICs (19/25) reporting mainly on national TB guidelines or policies. We identified barriers and facilitators across the SURE framework levels and domains. Most themes were reported in both LMICs and HI countries except poor-resourced facilities and those in the social and political constraints level which were only reported in LMICs. The main barriers were mistrust of tests and health providers, disbelief of diagnosis and stigma by recipients of care. At the providers of care level, limited awareness of guidelines, poor understanding of the disease, limited skills in sample collection, lack of confidence in tests, mistrust of guidelines, fear of misusing tests and patients’ reactions guiding practice were the main barriers across all settings. At the health system constraint level, the barriers were poorly adapted and updated guidelines, ambiguous guidelines, rigorous guideline methodology, poor advocacy, low adherence to guidelines, limited education, poor patient flow processes and poor facilities. Others were donor and WHO dependence and low national policy ownership. The main facilitators were the trust of doctors and willingness to test by the recipients of care, knowledge about disease and guidelines by the healthcare providers, national leadership support, political will, stakeholder engagement and availability of training tools and opportunities.

Whereas our review focused on implementing guidelines or policies about the diagnosis of TB, the other published qualitative [11] or mixed methods reviews [10, 12] focused on downstream barriers and facilitators on the TB test itself, TB control (prevention and care) and associated health system factors. Some reported findings in these other reviews were similar to our findings demonstrating similar perceptions and considerations encompassing the TB case [49] or care pathway. For instance, Brown and colleagues [11] published a qualitative evidence synthesis of 11 studies evaluating the barriers and enablers to implementing an individual test, the Gene Xpert TB test in LMICs. In contrast, our review focused on the implementation of guidelines for any form of TB testing, mainly from LMICs. They reported barriers mainly related to individual patient factors (patient costs and distance to health facilities) and health system factors (human and infrastructural resources, service coordination and implementation challenges and technical operational challenges).

A mixed methods review of all types of studies, including qualitative ones (*n*=65) by Conroy and colleagues [10], revealed that the most common barrier to implementing TB prevention and care guidelines in European countries was poor adherence to TB prevention and care guidelines related to inadequate knowledge and perceived usefulness by clinicians. A rapid qualitative evidence synthesis by Houghton and colleagues [13] exploring barriers and facilitators to adhering to Infection Prevention Control (IPC) guidelines for various respiratory infections pointed to several factors, including issues with ambiguous and poorly communicated guidelines, support for managers, workplace culture, training and access to equipment and facilities.

Health system constraints constituted most of the reported barriers in our review. Guidelines, if not well disseminated and implemented, will not impact healthcare practices [50]. The knowledge-to-action cycle proposed by Graham and colleagues [51] provides a valuable framework for planning dissemination and implementation activities that discuss tailored approaches based on an assessment of local or contextual barriers and facilitators. In addition to the main guideline, an additional toolkit on implementation attached to a guideline would help show end users how to effect the guideline recommendations. For example, the WHO consolidated guidelines on tuberculosis [52] had an accompanying operational handbook [53] or implementation guide. Gagliardi and colleagues [54] conducted a methodological review of 35 documents on guideline implementation and showed that none had instructions for operationalizing implementation strategies. From this, they developed a checklist for guideline implementation planning that guideline developers or policymakers could employ. Implementation plans should also be developed considering WHO’s Handbook of Health System Indicators and Measurement Strategies [55]. This handbook lists six main health system domains to be considered: service delivery, workforce, information systems, access to essential medicines (and diagnostics), financing, leadership and governance.

Poorly adapted and contextualized guidelines were some of the barriers identified in the implementation of guidelines. Effective guideline adaptation could facilitate better planning for implementation depending on the context. However, approaches lack clarity globally [56–58]. Surveys of guideline adaptation methods and frameworks have reported the use of different methods of varying quality, time-consuming methods and shortcomings in the evaluation of the adaptation process especially in low-income settings [58]. In addition, a survey of 72 articles reported that most published guidelines in peer-reviewed journals [56] did not report a published adapted method. Recent methods such as GRADE-ADOLOPMENT have been proposed and utilized in guidelines in high and middle-income settings [59–62]. However, to our knowledge, in addition to reports of its use, systematic evaluations of its use and impact have not been published. A case study of guideline adaptation in South Africa revealed that although most guideline recommendations originated from high-income countries, there were opportunities to strengthen guideline adaptation locally through stakeholder engagement to improve their uptake [57].

From our findings, the availability of training tools and opportunities, stakeholder engagement and knowledge about disease and guidelines by the healthcare providers enhanced the implementation and uptake of guidelines. A qualitative study by McCaul and colleagues [63] aimed to strengthen guideline uptake in South Africa by obtaining the perspectives of prehospital providers’ reported facilitators such as technology to support end-user documents, establishing online or modular guideline training and local champions to support change. Political will was also pointed out as a facilitator of TB guideline implementation from our review findings especially in LMICs. In recognition of the importance of political will in improving TB prevention and care, the WHO released a joint statement with the WHO’s Civil Society Taskforce on TB, calling for increased political commitment and accountability for TB [64]. Among the three key pillars of this statement was the need to support the rapid adoption of WHO guidelines at the country level.

The strengths of our review include an extensive search of literature in electronic sources and grey literature and reporting according to the ENTREQ statement. To minimize selection and reviewer bias, two review authors independently performed study identification and data extraction. Furthermore, most included studies were from LMICs where the burden of TB is high, and most studies reported on national guidelines or policies on TB diagnosis, which are most applicable or contextual compared to global policies. We also included barriers and facilitators as reported by a diverse group of health stakeholders, including recipients and providers of care. Our review was limited by having English-only studies; thus, studies from non-English high TB-burden countries were likely missed. Further, qualitative studies on barriers to implementation strategies targeting improvement in organizations (organizational culture, continuous quality improvement), healthcare workers (education, training, audit and feedback, reminders, patient-mediated interventions), and patients (reminders, financial incentives) were scarce. We only identified one study about a clinical audit [29]. Our review only identified studies from healthcare facility settings and did not identify evidence from community or home settings hence the results may only be generalized to health facility settings. Qualitative evidence provides supplemental insight to stakeholder’s experiences and preferences and should be interpreted alongside quantitative evidence on the same intervention or phenomenon.

Future research testing the effectiveness and processes of interventions to improve the implementation and uptake of TB testing guidelines would help provide an evidence base to plan and effect their implementation. Such user testing studies could include implementation trials, qualitative research and process evaluations targeting organizations, healthcare workers or patients. Social and political constraints were only reported in LMICs and it would be of interest to investigate the influence of these factors in HI countries on the implementation of TB diagnostic guidelines.

## Conclusion

Our review findings identified that the barriers to the implementation of guidelines for the diagnosis of TB are mainly about the limited test or guideline knowledge, trust and adherence related to poorly updated, developed, adapted and disseminated guidelines and poorly resourced facilities. Coupled with the resourcing of health facilities, improved training, adaptation, dissemination and advocacy of guidelines are likely to improve their implementation. Future research should consider the review findings on barriers and facilitators when designing approaches to implement TB guidelines.

### Supplementary Information

**Additional file 1.** Deviations from protocol.

**Additional file 2.** ENTREQ reporting checklist.

**Additional file 3.** The PRISMA checklist.

**Additional file 4.** The Search strategy.

**Additional file 5.** The SURE framework for identifying factors affecting the implementation of policy.

**Additional file 6.** The CASP quality assessment questions.

**Additional file 7.** List of excluded studies.

**Additional file 8.** CASP assessment results.

**Additional file 9.** CERQual Assessment of each barrier.

**Additional file 10.** CERQual Assessment of each facilitator.

**Additional file 11.** Barriers to implementation of TB diagnosis guidelines.

**Additional file 12.** Facilitators to the implementation of TB diagnosis guidelines.

## Data Availability

All data generated or analysed during this study are included in this published article.

## Abbreviations

ACF: Active case finding
CASP: Critical Appraisal Skills Programme
CDC: Centre for Disease Control and Prevention
CERQual: Confidence in the Evidence from Reviews of Qualitative Research
COVID 19: Coronavirus disease 2019
ENTREQ: Enhancing Transparency in Reporting Synthesis of Qualitative Research
HI: High income
LI: Low income
LMI: Lower middle income
LMIC: Low- and middle-income country
MTB/RIF: Mycobacterium tuberculosis/resistance to rifampicin
PRISMA: Preferred Reporting Items for Systematic Reviews and Meta-Analysis
PROSPERO: Prospective Register of Systematic Reviews
SURE: Supporting Use of Research Evidence
TB: Tuberculosis
UMI: Upper middle income
WHO: World Health Organization
